# Clinical bioinformatics for complex disorders: a schizophrenia case study

**DOI:** 10.1186/1471-2105-10-S12-S6

**Published:** 2009-10-15

**Authors:** Emanuel Schwarz, F Markus Leweke, Sabine Bahn, Pietro Liò

**Affiliations:** 1 Institute of Biotechnology, University of Cambridge, Tennis Court Road, Cambridge, CB2 1QT, UK; 2 Department of Psychiatry and Psychotherapy, University of Cologne, 50924 Cologne, Germany; 3 Central Institute of Mental Health, University of Heidelberg, Mannheim, Germany; 4 Computer Laboratory, University of Cambridge, 15 J.J. Thompson Avenue, Cambridge CB3 0FD, UK

## Abstract

**Background:**

In the diagnosis of complex diseases such as neurological pathologies, a wealth of clinical and molecular information is often available to help the interpretation. Yet, the pieces of information are usually considered in isolation and rarely integrated due to the lack of a sound statistical framework. This lack of integration results in the loss of valuable information about how disease associated factors act synergistically to cause the complex phenotype.

**Results:**

Here, we investigated complex psychiatric diseases as networks. The networks were used to integrate data originating from different profiling platforms. The weighted links in these networks capture the association between the analyzed factors and allow the quantification of their relevance for the pathology. The heterogeneity of the patient population was analyzed by clustering and graph theoretical procedures. We provided an estimate of the heterogeneity of the population of schizophrenia and detected a subgroup of patients featuring remarkable abnormalities in a network of serum primary fatty acid amides. We compared the stability of this molecular network in an extended dataset between schizophrenia and affective disorder patients and found more stable structures in the latter.

**Conclusion:**

We quantified robust associations between analytes measured with different profiling platforms as networks. The methodology allows the quantitative evaluation of the complexity of the disease. The identified disease patterns can then be further investigated with regards to their diagnostic utility or help in the prediction of novel therapeutic targets. The applied framework is able to enhance the understanding of complex psychiatric diseases, and may give novel insights into drug development and personalized medicine approaches.

## Background

Clinical bioinformatics is concerned with the analysis and visualization of complex medical datasets [1]. In contrast to the classical 'main stream' bioinformatics field which focuses on the analysis of biological information (See [2-4,1] for an introduction to Clinical Bioinformatics), here the main focus is to collate heterogeneous data sets from disparate data sources (e.g. patient clinical records, proteomics and transcriptomics data) and develop novel algorithms for the analysis of such heterogeneous data sets. Thus, the key goal is the simultaneous evaluation of clinical and basic research data with the aim to improve medical care and care provision (See [5] for data exploitation methods in cancer therapy development). For complex diseases such as psychiatric disorders, a wealth of information about patients is usually available. This includes clinical data, standard laboratory evaluations, genetic data, brain imaging data and data obtained from molecular profiling experiments. The recent advance in technological innovations allows to perform high throughput experiments resulting in an enormous increase in the amount of biomedical data generated. Yet, the different sources of data are commonly kept separate which means that valuable information is lost or neglected. Due to this lack of integrated analysis, the importance and relationships between clinical observations and the underlying molecular mechanisms are not understood. In clinical bioinformatics, a major aim is to combine these different sources of information and identify emerging features of the diseases under investigation. These features may reveal links to other pathologies and uncover networks of relationships between different diseases.

Novel clinical bioinformatics approaches could thus provide a better understanding and definition of complex diseases resulting in more accurate, improved diagnosis and better therapies. Over the last years, a need for personalized medicine is increasingly appreciated as it has been apparent that standard treatment approaches are rarely efficient across the entire patient population. Schizophrenia is a good example for a disorder that presents with a broad spectrum of different clinical manifestations which almost certainly is due to the existence of diverse underlying etiologies that happen to present clinically with similar symptoms [6]. Difficult diagnosis and low success of current drug regimes are an inevitable consequence. It would be highly desirable to identify patient subgroups corresponding exactly to the underlying disease pathology, thus facilitating the choice of the most appropriate treatment.

Here, we present a clinical bioinformatics approach to improve diagnosis and understanding of complex psychiatric diseases, which entails the application of a graph theoretical approach that captures information about patients and all disease associated data in a network. We investigate the relationships between patient specific variables and the disease and show how dependencies between these variables can be used to obtain important insights into disease pathologies and are directly related to improved diagnostic approaches. We use this methodology for an integrated assessment of data derived from different profiling platforms and standard laboratory tests and show how it can improve the understanding of highly heterogeneous disorders.

## Results and discussion

### Schizophrenia – a complex disease

The clinical data used in this study was derived from two different profiling platforms and standard laboratory tests. Metabolites in the CSF of 77 individuals (33 first episode drug naive patients suffering from acute schizophrenia (DSM-IV: 295.30 or 298.8) and 44 demographically matched healthy volunteers) were profiled by H-NMR. Serum proteins of the same subjects were investigated by LC mass spectrometry. For the NMR dataset, signals corresponding to the same molecules were averaged. In the mass spectrometric dataset, each variable referred to a mass spectrometric peak including adduct formation and isotopes. The study also includes measurements of CSF and serum glucose levels derived from a standard laboratory test on all patients.

**Figure 1:**
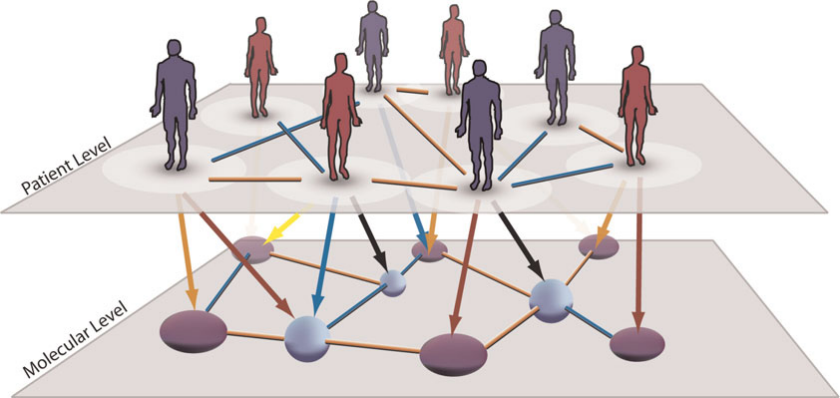
**The complex disease network**. Network representation of the information available in complex diseases. The top layer represents patient information and links between patients reflect associations between patients such as family relationships, sexual contact, ethical background or geographical proximity. The lower layer contains information about factors underlying the complex phenotype as quantified in genetic studies, molecular profiling experiments, clinical data etc. There is a strong dependency structure between the two layers that connect individual patients to abnormalities in the lower layer. The network concept thus allows the representation of available information in a patient specific manner.

The structure of the available data can be easily visualized in the form of two layers of a network (Figure 1). The first layer contains all patients and reflects the dependency structure between them. These dependencies may arise from common genetic backgrounds or similar endo-phenotypes. In the present study, all patients were unrelated. The second layer of the network comprises all variables assessed in the patient population. In the present study, these variables were levels of molecules in the serum and CSF of the patients. Similarly, the layer could contain information about the clinical state of the patients, e.g. symptom severity, blood scans, brain imaging results etc. The interactions in the lower layer reflect the dependency structures between the variables, for example involvement in related biochemical pathways.

Importantly, these associations may reveal disease relevant features such as dependencies between symptom severity and genetic background or levels of blood proteins. For psychiatric disorders, dependency structures such as the association between clinical features such as hallucinations or delusions and respective molecular abnormalities are not well understood.

**Figure 2:**
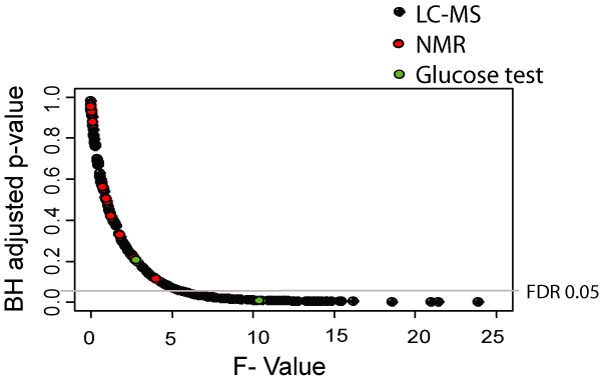
**Exploratory analysis – FANOVA**. F-values and FDR adjusted p-values of the variables contained in clinical dataset. Several variables remain significant after controlling the FDR.

In the first instance, we applied FANOVA to investigate differences between the patient and control populations. In clinical terms, this means to identify which individual factors are associated with the disease state. To correct for multiple hypothesis testing, the False Discovery Rate was controlled and all p-values adjusted accordingly (Figure 2). The determination of the relationship between individual molecules and the disease state embodies an intuitive connection between exploratory data analysis and the investigation of dependencies between variables. In complex diseases, interpretation is greatly improved if associations between disease associated variables are known and sometimes correlated analytes can be of great help to uncover abnormal pathway structures. We were able to identify several variables that remained significant after controlling the false discovery rate at a level of 0.05.

**Figure 3:**
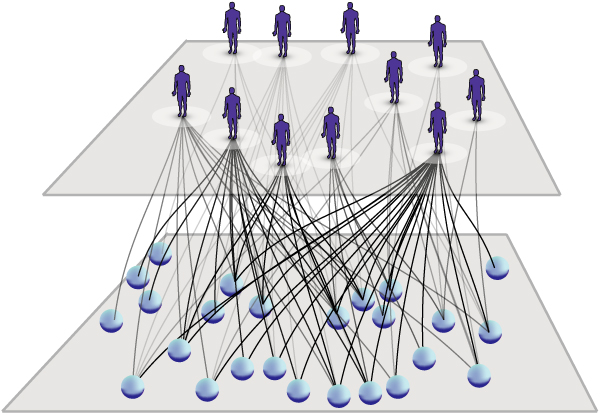
**Disease network of a patient subgroup**. The network reflects a remarkable abnormality in serum levels of primary fatty acid amides found in a subgroup of patients.

We aimed to use the links in the two-layer network to assess the complexity of the disease by determining whether all abnormalities were equally distributed amongst the patient population or whether these abnormalities are concentrated in particular systems of molecules or subgroups of patients. Therefore, similar to Barabasi et al. [7], a bipartite network was generated that included all molecular analytes in one partition and all patients in the other partition. We assessed the heterogeneity in the network by Markov chain clustering. The algorithm was expected to cluster patients that featured similar abnormalities with the affected molecular compounds. The clustering of patients and variables gives, therefore, an estimation about the heterogeneity of information in the network. Maximization of the bipartite modularity yielded 195 distinct clusters. One third of the patients could be found in one cluster having profound abnormalities in levels of serum primary fatty acid amides which were not shared by the remaining patients (Figure 3). The identity of the primary fatty acid amides was confirmed by re-analysis of commercially available standard compounds.

**Figure 4:**
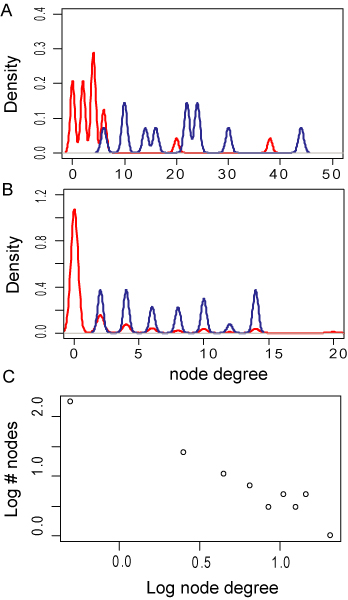
**Degree distributions of patient and molecular networks**. Bipartite modularity maximization guided by Markov Clustering algorithm determined a subgroup of patients (panel A) that hat highly increased node degrees (blue line) as compared to the remaining patients (red line). The associated molecules found in the same cluster (blue line, panel B) featured the same increased node degree. The node degree of the molecular analytes was found to follow a power law distribution (panel C).

The heterogeneity of the patient population is well reflected in the distribution of the node degrees. It can be seen that the patients co-clustering with the network of primary fatty acid amides have a far higher average node degree than the remaining patients (Figure 4A). The same phenomenon can be observed for the primary fatty acid amides which have a far greater average node degree than the remaining molecules (Figure 4B). It can also be seen that the degree distribution of the molecular compounds follows power law indicating the presence of few highly connected nodes (Figure 4C).

Interestingly, the abnormalities found for primary fatty acid amides were not related to the significant differences we observed in CSF Glucose and glutamate levels which are a known feature of the schizophrenia pathology [8].

**Figure 5:**
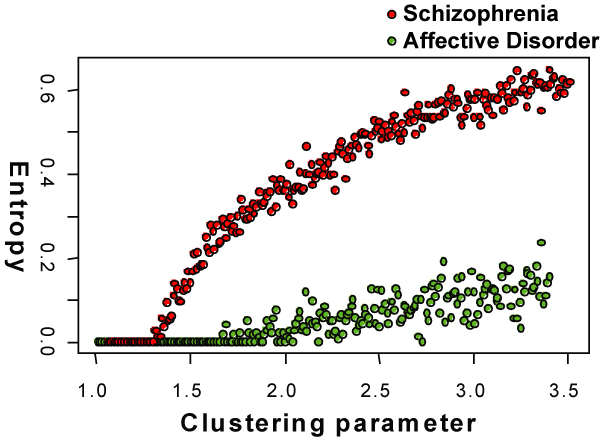
**Stability of Networks in schizophrenia and affective disorder**. Comparison of the stability of the primary fatty acid networks determined from schizophrenia and affective disorder patients. The stability is measured using the networks' entropy during the clustering procedure. The entropy decreased at a lower rate in affective disorder, reflecting higher stability and a stronger alteration of the primary fatty acid network.

In an extended dataset (70 antipsychotic naive schizophrenia patients, 39 affective disorder patients and 59 healthy individuals; considering mass spectrometric data only), we confirmed the dependency structure of the above mentioned fatty acid network and compared the associations between schizophrenia and affective disorder. Figure 5 compares the stability of the fatty acid network using the entropy measure during the clustering procedure. For schizophrenia patients, the entropy increased at a lower clustering coefficient and followed a log-linear shape. For affective disorder patients, the network was more stable and split apart at a higher clustering coefficient. The shape of the entropy curve was linear for affective disorder patients and reached a far lower value than the network derived from schizophrenia patients. In affective disorder, the network of primary fatty acid amides was strongly connected and very stable due to a higher degree of alteration of the molecules in this patient group.

### Mapping patients on the "diseasome" network

The concept of encoding relationships between individuals, diseases or molecules into networks is called network medicine [9] and has been shown to produce biologically interesting results in the investigation of disease gene associations [7]. As noted by Loscalzo et al, the application of network concepts in complex diseases can result in novel approaches for diagnosis and treatment [10]. Here, we applied this concept to achieve an integrated representation of complex disorders encoding all relevant information simultaneously. Graph theoretical approaches are suited to capture the complexity of human diseases and provide a theoretical framework to easily incorporate molecular readouts and patient information to give a comprehensive description of a disease state.

Using the graph theoretical approach, the similarity of patients can be readily determined from the integrated patient information enabling the assessment of disease similarity and possibly, the subclassification of patients. This would be particularly desirable for psychiatric disorders for which the highly heterogeneous symptoms may result from different etiologies and possibly contribute to the low efficacy of current drug regimes. Extending the concept of subclassifying patient cohorts to the single patient level leads to a conceptual framework often referred to as personalized medicine. Patient specific information can be incorporated into the network approach and may allow for an individualized assessment of a given patient's disease state [10]. Besides facilitating more efficient treatment approaches, a system of robust yet patient specific hallmarks of a complex disorder would be invaluable in the design of clinical trials, the development of new drug candidates or the identification of novel drug targets. In the context of psychiatric disorders, a personalized diagnosis and treatment approach would be of particular value as patients' responsiveness to treatment can currently not be predicted, impeding appropriate and successful therapy.

**Figure 6:**
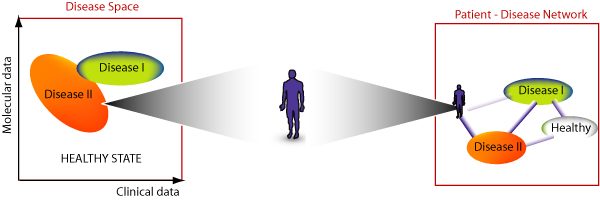
**Mapping patients on the "diseasome" network**. The spectrum of available data in complex diseases can be imagined as forming a "disease space" within which every disease occupies a particular location (left panel). In terms of networks, each disease forms a node and related diseases are connected by links reflecting the strength of association. The diagnostic process can be imagined as mapping a patient on the disease space or the "diseasome" network.

Networks are an intuitive concept of visualizing disease related information. Importantly, they may allow the investigation of relationships between related diseases such as schizophrenia and depression which may share parts of their biochemical underpinnings. Often, the consideration of clinical information only is not sufficient to diagnose these diseases unambiguously. In fact, no single symptom is specific for schizophrenia [6]. The presence of certain symptoms or molecular abnormalities ultimately results in the diagnosis of a certain disease. In other words, the spectra of symptoms and other possible abnormalities such as molecular alterations form a 'disease space' in which every illness has a particular location (Figure 6). It is important to note that finding a patients location in this disease space is by no means bound to be a 'black and white' process in which a particular individual either has a disease or no disease. We would rather be able to replace this binary thinking by a continuous scale in which intermediate states between being ill and being healthy are possible. From a network point of view, the disease space can be imagined as a graph in which the diseases form the nodes and the relatedness between them the links. Finding the patient's exact location in this network or disease space is at the heart of personalized medicine and has many important implications for treatment approaches as a treatment of the patient ultimately results in a 'movement' of the patient across the network. Mathematical formalization will be described in a separate manuscript.

## Conclusion

In this study, we analyzed complex psychiatric diseases in the form of disease networks. We quantified robust associations between analytes measured with different profiling platforms and standard laboratory tests and were able to determine a subgroup of patients that featured remarkable abnormality in a molecular system of primary fatty acid amides. The results were validated in an extended dataset of schizophrenia patients and the network properties compared to the ones present in affective disorder. We found that in affective disorder, the molecular networks were more profoundly altered when compared to schizophrenia. The methodology helps to statistically assess the complexity of a given disease and disease associated patterns can then be further investigated with regards to their diagnostic utility or help in the prediction of novel therapeutic targets. The applied framework is able to enhance the understanding of complex psychiatric diseases and may give valuable insights into drug development and personalized medicine approaches.

## Methods

### State of the arts methods in clinical bioinformatics

There is currently a tremendous growth in the amount of life science high through-put data which has been paralleled by similar growths of electronic storage of clinical data. Bioinformatics is experiencing a period of great capability in providing the methodologies complementing life science experimental research and they are keeping the pace with the growing availability of a variety of molecular biology high through-put data. Physicians and biologists are now pressing with more challenging requests. The most important issue is about the integration of the different types of high throughput data (omics), the second is the integration of molecular biology data with clinical data. The integration of such large heterogeneous amount of data is representing the start of a new golden age for artificial intelligence and in particular for machine learning techniques related to clinical bioinformatics.

In clinical bioinformatics for complex diseases, data from multiple sources are integrated constituting a multiscale challenge. Data integration on large scale datasets has been successfully applied for gene function prediction (see [11] and references therein). A different approach tried to derive more informative data from heterogeneous datasets by means of consensus clustering [12,13]. In the clinical context, a kernel method based on Support Vector Machines was introduced to combine microarray data with clinical data for diagnosing breast cancer patients [14]. The method has recently been applied for the combination of proteomics and microarray datasets derived from rectal cancer patients [15]. So far, methods used in clinical bioinformatics approaches focussed on the improvement of predictive power by integrating additional information.

Here, we follow a different approach by setting up a comprehensive analysis framework reaching from the initial stage of consistent data collection to integrated disease investigation. The basic procedure is as follows: First, we combine data from disparate sources such as molecular, clinical or phenotypological data into a compound dataset. Then, exploratory data analysis is performed to determine the relevance of single variables for a given pathology. If significant dependencies are found, we proceed towards an investigation of these relationships by means of graph theory and clustering methods. These methods were applied to determine robust, disease associated patters or molecular/clinical abnormalities. Such patterns can then be used to determine the disease state of a given individual, e.g. to assist diagnosis or evaluate treatment success [10].

### Statistics for complex diseases

Given the compound data matrix, we investigate the importance of single variables for the disease by means of ANOVA which is one of the most widely used tools in applied statistics. Functional analysis of variance (FANOVA) is a functional data analysis (FDA) form of the classical ANOVA [16-18] which has a strong link to multivariate analyses, such as principal component analysis, and to multivariate linear modeling and regularization methods that assume a particular class of smooth functions for the estimators. We give a brief description of the technique we implemented. Let *Y*_*ij*_(*t*) denote the signal *j *in the *i*-th experiment. Observations are modeled by a fixed effect ANOVA model(1)

where *i *= 1,....,*p*, *l *= 1,....,*n*_*i*_; , *u*(·) is the grand mean, *a*_*i*_(·) is the deviation of the mean in experiment *i *from the grand mean, and *e*_*ij*_(·) are i.i.d. zero-mean normal random variables with variance *σ*^2^. Here we use standard functional ANOVA setting as in [18]. Following the standard ANOVA treatment [18], for each t step, the function(2)

is distributed as non central(3)

This powerful methodology is quite general and would be ideal for preliminary data exploration of the meaningful variables for the different disease phenotypes. In our case, the F statistics gives us information about the link between measurement response and disease phenotype.

Caution has to be taken to account for multiplicity problems as it gets increasingly likely to determine significant F statistics as the number of investigated variables increases. Multiple methods exist to adjust for multiple hypothesis testing. A widely accepted method is the control of the False Discovery Rate (FDR) which controls the ratio of false positive findings among all rejected hypotheses [19]. This procedure is more powerful than more classical approaches such as the bonferroni correction, as it based on the rationale that few false positive findings are not too problematic if the number of positive findings is high.

Furthermore, FDR procedures do not assume that variables are independent and in fact, is has been proven that the FDR procedure is applicable to datasets containing dependencies between the variables [20]. Here we adjust p-values resulting from FANOVA using the FDR procedure suggested by Benjamini and Hochberg [19]. If single features were significant after the multiplicity adjustment, dependencies between the features were investigated. The dependency structure contains valuable information about the relationships between the investigated variables that is not apparent from exploratory analysis.

To analyze the dependencies between the different variables, we first encoded the data matrix into a directed graph of N patients and M molecules. Here, every patient *n*_*i *_is connected to a variable *m*_*j *_if this patient has an abnormal state of the variable. The 'abnormality' of the state of a variable is defined with respect to the distribution of the same variable in the control population and a link between a patient and a variable was only built if the value of the variable was outside three standard deviations of the control mean. This procedure generated a directed graph in which one partition contained all variables and the other partition all patients.

In the present study, we use clustering procedures on the directed graph to investigate the dependencies between molecular compounds. Based on the directed graph, a graph with m nodes can be constructed that reflects which variables are altered in patients simultaneously. This procedure is performed for all pairs of variables setting the weight of the respective links equal to the number of patients in which the variables have abnormal levels. The resulting graph contains information about the joint relevance of the variables for the disease state.

### Network analysis

We use a clustering algorithm that does not need information on the number of clusters which is often unknown in large-scale comparisons. Although there is now a wide range of clustering algorithms, only a restricted number can successfully handle a network with the complete and weighted graph properties. Among them, we cite the recent method proposed by [21] that is based on simulated annealing to obtain clustering by direct maximization of the modularity. The modularity has been introduced by [22] and it is a measure of the difference between the number of links inside a given module and the expected value for a randomized graph of the same size and degree distribution. The modularity Q of a partition of a network is defined as  where the sum is over all modules of the partition. *l*_*s *_and *d*_*s *_describe the number of links and the total degree of the nodes inside module s and L the total number of links of the network [23]. In a recent work on resolution limits in community detection [23] the authors give evidence that modularity optimization may fail to identify modules smaller than a certain scale, depending on the total number of links in the network and on the number of connections between the clusters. Because of its properties, at the end, we implemented the Markov Clustering Algorithm (MCL, [24]). Its input is a stochastic matrix where each element is the probability of a transition between adjacent nodes. The weights between *m*_*i *_and *m*_*j *_were given the frequency of variables *m*_*i *_and *m*_*j *_being altered, i.e. an abnormality of molecule *m*_*j *_recorded in patient *m*_*i*_.

For the clustering of the bipartite network, we incorporated the modularity measure into the MCL algorithm. The result of the clustering procedure is largely dominated by the choice of the contraction parameter *r*; low values of *r *result in large clusters whereas the network is decomposed into single nodes at high values of *r*. For each arising cluster, we increased *r *until the cluster was split into at least two sub-clusters; we then used the modularity of a bipartite network [25] to compare whether the split increased the modularity across all clusters or not. If the modularity was improved, the clustering procedure was continued at the respective community; otherwise it was continued at the next community until no cluster remained.

We modified the Java version of the MCL algorithm [26] to include the strategy of Gfeller et al., [27] which allow detecting unstable nodes and compare results obtained with different contraction parameters. In this algorithm, the starting matrix is modified to produce a novel matrix with a certain amount of noise added. The noise is homogeneously distributed between -*σw*_*ij *_and *σw*_*ij *_where *w*_*ij *_is the edge weight and *σ *a fixed noise parameter, 0 ≤ *σ *≤ 1. The noise was added randomly to edges and the MCL clustering was performed on many noisy realization of the matrix. At each 'noisy' repetition, the algorithm recorded all the nodes belonging to the same cluster. After the prefixed number of repetitions has been concluded we ended up with a matrix storing *P*_*ij *_values corresponding to the fraction of times nodes *i *and *j *have been grouped together. Unstable nodes can be identified as those having edges with less than a fixed values *θ*. We then calculated several distinct measures informing on the clustering and its stability such as the following clustering entropy:(4)

where the sum is over all edges and the entropy is normalized by the total number of edges, *L *[28]. This might be used to detect the best clustering obtained after a long series of clusterings with different granularity parameters each time.

The entropy can also be used to study the stability of communities obtained from the clustering procedures. Due to the repeated noisy realizations of the original matrix, nodes may be attached to different communities after the clustering procedure. However, if the investigated system is very stable, nodes tend to cluster with the same communities regardless of the added noise. The stability of the different communities can be investigated by analyzing the entropy as a function of the clustering parameter r as the network breaks down into increasingly separated clusters as r increases.

### Schizophrenia data used in this study

In this study, Cerebrospinal Fluid (CSF)and serum samples from a large cohort of 77 individuals were used (for a detailed characterization of the patient population see [29]). CSF surrounds the brain and is besides its functions regarding mechanical protection, a transport medium for important molecules. Due to its close proximity to the brain, it is likely that pathological abnormalities of the brain are reflected in the CSF. Serum samples most body tissues and fluids and is also an important carrier of signalling molecules. In both serum and CSF samples, a global metabolic profiling was conducted. CSF samples were profiled using proton NMR spectroscopy, serum samples using Liquid Chromatography Mass Spectrometry. The profiled samples (CSF and serum) included 33 samples from drug naive first onset schizophrenia patients and 44 samples taken from healthy volunteers. In an extended analysis we profiled serum samples from 91 additional samples comprising 33 antipsychotic naive first onset schizophrenia patients, 39 samples obtained from patients suffering from affective disorder and 15 controls. We assessed glucose concentrations in the serum and CSF of all individuals and integrated the information with mass spectrometric and NMR data. The ethical committees of the Medical Faculty of the University of Cologne approved the protocols of this study. Informed consent was given in writing by all participants and clinical investigations were conducted according to the principles expressed in the Declaration of Helsinki.

## Competing interests

The authors declare that they have no competing interests.

## Authors' contributions

E-S, P-L and S-B designed the experiments for this study. E-S performed the mass spectrometric experiments, statistical analysis and wrote the manuscript. FM-L collected the biological samples. All authors read and approved the final manuscript. Please contact P.L. for bioinformatics and S.B. for clinical/scientific correspondence
